# Challenges in modelling the random structure correctly in growth mixture models and the impact this has on model mixtures

**DOI:** 10.1017/S2040174414000130

**Published:** 2014-03-03

**Authors:** M. S. Gilthorpe, D. L. Dahly, Y.-K. Tu, L. D. Kubzansky, E. Goodman

**Affiliations:** 1 Division of Epidemiology & Biostatistics, School of Medicine, University of Leeds, Leeds, UK; 2 Department of Epidemiology and Public Health, University College Cork, Cork, Ireland; 3 Institute of Epidemiology & Preventive Medicine, College of Public Health, National Taiwan University, Taipei, Taiwan; 4 Department of Social and Behavioral Sciences, Harvard School of Public Health, Boston, MA, USA; 5 Mass General Hospital for Children, Department of Pediatrics, Harvard Medical School, Boston, MA, USA

**Keywords:** autocorrelation, growth, mixtures, random effects

## Abstract

Lifecourse trajectories of clinical or anthropological attributes are useful for identifying how our early-life experiences influence later-life morbidity and mortality. Researchers often use growth mixture models (GMMs) to estimate such phenomena. It is common to place constrains on the random part of the GMM to improve parsimony or to aid convergence, but this can lead to an autoregressive structure that distorts the nature of the mixtures and subsequent model interpretation. This is especially true if changes in the outcome within individuals are gradual compared with the magnitude of differences between individuals. This is not widely appreciated, nor is its impact well understood. Using repeat measures of body mass index (BMI) for 1528 US adolescents, we estimated GMMs that required variance–covariance constraints to attain convergence. We contrasted constrained models with and without an autocorrelation structure to assess the impact this had on the ideal number of latent classes, their size and composition. We also contrasted model options using simulations. When the GMM variance–covariance structure was constrained, a within-class autocorrelation structure emerged. When not modelled explicitly, this led to poorer model fit and models that differed substantially in the ideal number of latent classes, as well as class size and composition. Failure to carefully consider the random structure of data within a GMM framework may lead to erroneous model inferences, especially for outcomes with greater within-person than between-person homogeneity, such as BMI. It is crucial to reflect on the underlying data generation processes when building such models.

## Background

Lifecourse researchers often estimate growth curves or ‘trajectories’ in longitudinal data to understand developmental processes. Multilevel modelling^1^ ^,^ ^2^ is perhaps the most popular method of growth curve estimation in health research, but other useful methods based on structural equation modelling^3^ are more commonly used in the social sciences. These include latent growth curve modelling (LGCM)^4^ ^–^ ^7^ and growth mixture modelling (GMM).^8^ ^–^ ^17^ In LGCM, repeated measures of a growth variable (e.g. height) are modelled as a function of a smaller number of latent growth factors (analogous to the random effects of multilevel models) and time-specific latent errors. The latent growth factors and errors are each assumed to be independent and identically normally distributed, and the parameters of this ‘random structure’ help describe a mean trajectory in the population and how individuals deviate from that trajectory. GMM can be viewed as an extension of LGCM, where model parameters are allowed to vary across a specified number of latent classes.

In seeking a suitable standard GMM, it is currently common practice to estimate multiple models, specifying a different number of latent classes, and make a decision on which model is ‘best’. Individuals are classified by estimating their posterior probabilities of class membership. When a GMM with 2+ latent classes is a better explanation of the observed data than a single class model, it suggests that the population comprises sub-groups, each with its own underlying developmental process. Sub-group membership is interpreted as an important feature in its own right, related to health outcomes and other important covariates. Selecting the model with the ‘correct’ number of latent classes is central to GMM interpretation, and selection can be heavily influenced by the method used to parameterize the structure of random effects within the model. For example, a common approach is to constrain the growth factor variances of all latent classes to be zero, referred to as latent class growth analysis,^18^ group-based trajectory modelling^19^ or semi-parametric growth modelling.^20^ At the other extreme of model parsimony, one could freely estimate the variances and covariances of the growth factors separately for each latent class. It is also common to specify homoscedastic or heteroscedastic models by constraining or freely estimating the latent error variances across time points and/or classes.

Choices regarding model parameterization should be driven by an understanding of underlying data generation processes, associated theory and the research question at hand. GMM convergence can be difficult when there are too many freely estimated parameters. A common solution is to simplify the model with parameter constraints. Although these constraints might be necessary for model estimation, they may not accurately reflect the underlying growth process, and can thus lead to erroneous conclusions. When growth factor variances and covariances are constrained to be zero, autocorrelation among the time-specific latent errors emerges. This occurs if individual growth curves are regularly above or below the class-specific mean growth curve, which is likely for large parts of an individual growth trajectory if outcomes exhibit more between-subject than within-subject heterogeneity (which is the case for most human growth measures). This might be resolved by freely estimating the growth factor variances and covariances; however, as noted above, such free estimation may be impossible because of convergence problems. An alternative, more parsimonious approach is to model explicitly the emergent autocorrelation structure. To date, no study has examined the impact of doing this on the selection and interpretation of GMMs. Our study addresses this gap. We consider the simple approach of imposing an autocorrelation constraint on successive measures.

Body mass index (BMI) is a variable of great interest to researchers in a variety of fields and has been studied previously using GMM.^8^ ^,^ ^10^ ^,^ ^12^ ^,^ ^14^ ^,^ ^16^ We use a motivating example of exploring lifecourse patterns of BMI in a sample of adolescents. Prior work with this cohort has assessed cardiometabolic risk, psychological distress and weight status.^21^ ^–^ ^23^ In our illustration, we use these data to generate GMMs to identify lifecourse patterns of BMI, while considering different model parameterizations. We also simulate BMI growth data for a simple model in the same context to help inform interpretation of the findings from the genuine data. We contrast constrained models with and without an autocorrelation structure, to reveal the impact this has on the derived model, specifically the ideal number of latent classes, their size and their composition. Simulations inform how constraining a GMM’s random structure can introduce an emergent autocorrelation structure, and how failure to model this explicitly can lead to erroneous models being selected.

## Methods

### The study data set

This study uses longitudinal data from a cohort study conducted in Cincinnati, OH, US area.^21^ ^–^ ^23^ Data were drawn from Phase 1 of the Princeton City School District study, which began in the 2001–2002 school year and included students in grades 5–12 at baseline with three further annual waves of data collection. Students were excluded if they were pregnant, received corticosteroid treatment for asthma, had a disease that would interfere with carbohydrate metabolism (diabetes, cancer, cystic fibrosis, acromegaly, Cushing’s disease or syndrome, pheochromocytoma, liver or kidney disease) or were participating in a longitudinal study of carbohydrate metabolism. Study visits included a physical exam where height and weight were measured. As the cohort was 95% non-Hispanic black and white, analyses were restricted to these two ethnic groups.

### Statistical methods

GMM and data simulation were carried out in Mplus version 7^24^ using maximum likelihood (ML) estimation to identify sub-groups that deviated from the ‘normal’ adolescent BMI trajectory. We modelled cohort trajectories (i.e. students nested within measurement occasions, irrespective of their ages) rather than age-specific growth trajectories (which would overlook the natural cohort clustering), as this accurately reflected the structure of the data.

BMI trajectories were taken to be quadratic in (centred) time, requiring three latent growth factors, hereby referred to as the intercept, velocity and acceleration. The intercept was modelled conditional on age at the first measurement occasion, sex, an age–sex product interaction term and racial/ethnic group. Covariate coefficients were constrained to be identical to ensure that the parameterization of underlying BMI growth curves was identical across classes. Individual trajectory differences in *mean* BMI by age, sex, age–sex interaction and racial/ethnic group were thus accommodated, as were the different ages at which students were recruited. The age–sex interaction allowed for mean BMI sex differences to vary according to age, and vice versa, accounting for growth spurt differences. As the underlying age, sex and racial/ethnic differences in mean BMI across the classes throughout adolescence were modelled, ‘residual’ differences amount to individual deviations from the underlying mean. Similarly, velocity and acceleration were conditional on age, allowing for differences in *change* in BMI by age throughout adolescence. Age and sex differences in BMI changes were captured via the age–sex interaction for the intercept. As initial investigation for age and sex interactions with race revealed small, non-significant coefficients, differences in BMI changes by racial/ethnic group were not modelled. As outcome variances appeared consistent over time, measurement occasion-specific variances were constrained to be identical across waves within each class (i.e. homoscedasticity was assumed).

Models of the illustrative data with freely estimated variance–covariance structures (i.e. random intercept, velocity and acceleration for each class trajectory) often gave rise to a non-positive definite covariance matrix, which led to difficulties in convergence. This is not unusual with such models. A common solution to this problem is to constrain some of the variance–covariance parameters to be zero. For our data, variances of both velocity and acceleration had to be constrained to be zero before models consistently converged. Details of the model specification for the illustrative data set are given in the Supplementary material.

Model-fit criteria examined were: −2 log-likelihood (−2LL); the Akaike’s Information Criterion (AIC=−2LL+2*k*, where *k* is the number of model parameters); and the Bayesian Information Criterion (BIC=−2LL+*k*×ln[*n*], where *k* is the number of model parameters and *n* is the sample size). The −2LL improves asymptotically towards model saturation with increasing model complexity, whereas the inclusion of penalty terms in AIC and BIC attenuate this, both seeking parsimony. Consequently, AIC and BIC can attain minima for relatively low values of *k*. Either AIC or BIC may be preferred in pursuing model parsimony, but one must remain mindful of the impact of parameterization on the utility and meaning of the GMM adopted.

Selection of the ‘ideal’ number of latent classes should be a combination of likelihood-based model-fit criteria and interpretational value. The number of latent classes that we examined for the illustrative data set ranged from 2 to 11. As the risk of models converging to local minima increases with increasing number of classes,^25^ models were run for 20k random starts (for a limited number of iterations), of which the best 10% (according to ranked LL) were run to completion to derive final model estimates; the number of converged models was examined to determine what proportion settled on the same ML value.

To evaluate whether models that differed only with regard to the parameterization of autocorrelation had the same individuals allocated to classes, we ranked classes by size for each model type and assessed class ‘correspondence’ for modal assignment. We used the Rand statistic for cross-classification agreement,^26^ the adjusted Rand statistic which accounts for chance,^27^ Stuart’s test for homogeneity,^28^ and a summary measure of *net drift* of the class membership from larger to smaller classes between models with and without AR1 structure.

Means of residual variances within each class were calculated for all models, weighted according to class size, yielding a measure of within-class random intercept heterogeneity. The overall BMI trajectory intercept variance (constrained to be identical across all classes) provides a measure of between-class random intercept heterogeneity. Both measures reflect how the random structure is partitioned within and between classes for each parameterization.

To inform interpretation of the findings from the illustrative data set, Monte Carlo simulations were undertaken using parameters guided by the genuine data. Details of the model specification for the simulated data are given in the online appendix. Simulated BMI growth data were evaluated using three GMM parameterizations: (a) unrestricted random effects (reflecting the underlying data generation process); (b) restricted random effects comprising random intercept only and no covariance terms (as per the constraints adopted to aid convergence); and (c) identical restricted random effects plus AR1 [a more parsimonious alternative to the unrestricted random effects that captures the emergent autoregressive (AR) structure]. Models were run for 10k random starts, of which the best 10% were used to derive model estimates. These were summarized over all viable replicates that attained convergence. For parameterizations (b) and (c), models were explored for a number of latent classes to explore changes in model likelihood statistics. Class composition was investigated for a subset of common replicate data sets where convergence was achieved for all parameterizations.

## Results

Demographics of the illustrative study in relation to BMI are summarized in Table 1. The cohort was 51.2% female, 47.1% black and had mean age at baseline of 14.4 years (sd=2.1). There were no substantive differences by age, sex or ethnicity between the 1528 students who completed two or more study visits (data used for this study) and the 222 who did not (data omitted from this study). All students had BMI assessed at baseline, 78% had a BMI assessment at all four waves, 15% had BMI assessment at three waves and 7% had a BMI assessment at two waves.

**Table 1:** Study data set structure and features

	*n* (%)	Mean BMI (s.d.)
Gender		
Male	745 (48.8)	23.0 (5.6)
Female	783 (51.2)	23.7 (6.3)
Race/ethnicity		
White	809 (52.9)	22.5 (5.0)
Black	719 (47.1)	24.4 (6.8)
Pubertal status		
Puberty	749 (49.1)	21.9 (5.5)
Post-puberty	776 (50.9)	24.8 (6.1)
Parent’s education		
<High school	358 (23.4)	23.8 (6.4)
High school	447 (29.3)	24.4 (6.9)
Some college	419 (27.4)	23.0 (5.3)
College or more	304 (19.9)	21.9 (4.3)

BMI, body mass index.

A summary of all models explored for the illustrative data, convergence characteristics and model-fit criteria are given in Table 2.

**Table 2:** Summary of growth mixture model (GMM) convergence characteristics and model-fit criteria for the illustrative study data: 10 restricted standard GMMs (Std) and 10 restricted AR1 GMMs (AR1)

	Convergence		-2LL		AIC	BIC	
	% success^a^	% agree^b^	Estimate	df	Estimate	Estimate	AR1(ρ)
Std							
2-class	100.0	100.0	24,235.8	15	24,265.8	24,345.8	–
3-class	100.0	100.0	23,742.2	20	23,782.2	23,888.9	–
4-class	99.9	100.0	23,396.8	25	23,446.8	23,580.1	–
5-class	99.8	90.8	23,140.8	30	23,200.8	23,360.7	–
6-class	99.8	60.5	22,949.8	35	23,019.8	23,206.4	–
7-class	99.6	100.0	22,841.5	40	22,921.5	23,134.8	–
8-class	99.4	100.0	22,758.2	45	22,848.2	23,088.1	–
9-class	99.1	28.9	22,699.9	50	22,799.9	23,066.5	–
10-class	98.9	10.7	22,656.3	55	22,766.3	23,059.5	–
11-class	98.7	11.5	22,617.1	60	22,737.1	23,057.0	–
AR1							
2-class^c^	19.7	100.0	22,916.2	15	22,946.2	23,026.2	0.944
3-class	20.1	100.0	22,692.4	21	22,734.4	22,846.3	0.923
4-class	20.1	26.1	22,613.6	26	22,665.6	22,804.3	0.873
5-class	20.1	23.4	22,561.9	31	22,623.9	22,789.2	0.842
6-class	19.4	48.1	22,522.8	36	22,594.8	22,786.8	0.850
7-class	19.5	46.3	22,499.2	41	22,581.2	22,799.8	0.805
8-class	19.7	8.7	22,472.1	46	22,564.1	22,809.3	0.733
9-class	19.7	1.4	22,450.7	51	22,552.7	22,824.6	0.734
10-class	19.4	1.9	22,432.0	56	22,544.0	22,842.6	0.742
11-class	19.4	0.2	22,414.4	61	22,536.4	22,861.6	0.742

LL, log-likelihood; AIC, Akaike’s Information Criterion; BIC, Bayesian Information Criterion; df, degrees of freedom.

a Percentage of successes derived as proportion of the 20k random starts that converged to a maximum likelihood.

b Percentage of successes as proportion of the 2k (10%) better models that converged that also agree on the same log-likelihood value derived.

c For the two-class AR1 model, the intercept variance was constrained to zero to attain convergence with non-negative variances.

### Model convergence

Almost all random starts converged for models with no AR1 structure, although the proportion of the best 10% that settled on the same ML value varied, with greater consistency observed for models with two to four latent classes or models with seven and eight classes. Among models with an AR1 structure, only 20% of random starts converged, indicating a limited solution space for models with this random effects parameterization; that is, many random starts began too far from a viable solution and many more random starts were needed to conduct an exhaustive search for potential solutions. Although one may predetermine starting values, the default is to permit randomly generated initial values. Among the best 10% of models that converged, consistency in the optimum ML varied, but was generally a smaller proportion than for models with no AR1 structure: ML agreement reduced markedly from 100% for the two- and three-class models to 0.2% for the 11-class model.

### Likelihood-based model-fit criteria and optimum number of classes

**Fig. 1:**
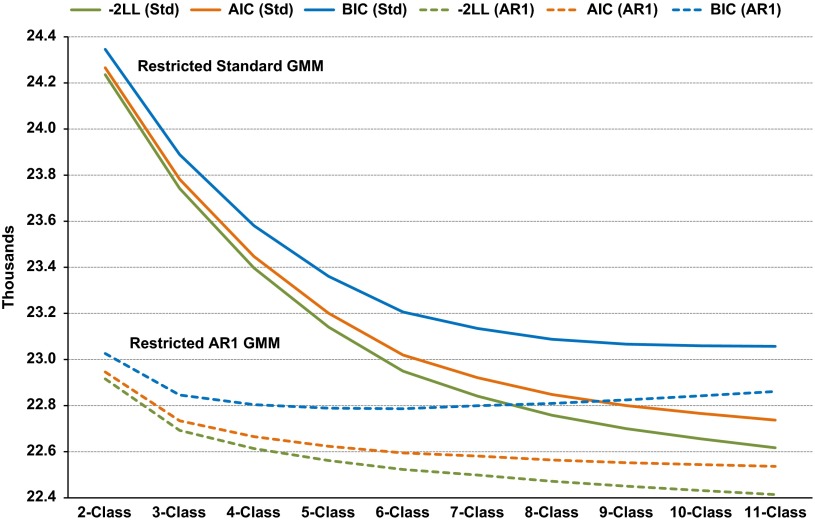
Likelihood-based model-fit criteria for growth mixture models (GMMs): 10 restricted standard (Std) and 10 restricted AR1 (AR1).

A graphical summary of the likelihood-based model-fit criteria is presented in Figure 1. The BIC favoured less complex models over AIC, as anticipated. Models with AR1 consistently fitted better than those without. For these particular cohort data, AIC and BIC never attained a minimum up to the 11 latent classes considered for models without AR1; BIC plateaued around 10 or 11 classes. A minimum BIC occurred at six classes for models with AR1.

### Class size and composition

Under the null hypothesis of no class discordance between models with or without AR1, class sizes should remain the same and classes ranked by size should correspond to the same class across both model types, with the ideal that class membership corresponds 100%. In practice, although correspondence between models generally decreased smoothly, there were three outlying values for the three-class, six-class and nine-class models when contrasted using modal assignment. The Rand statistic was optimistic, whereas the adjusted Rand, which accommodated chance, suggested modal agreement was often below 50% and near zero for the 3-class, 9-class and 11-class models. For models with three or more classes, there was a net *drift* of membership from smaller to larger classes with AR1 incorporated, and this was typically significant at the 0.1% level according to Stuart’s test, apart from the three-class and seven-class models (Table 3).

**Table 3:** Contrast of class correspondence based on ordered class sizes for 10 growth mixture models (GMMs) with and without AR1 based on modal assignment of 1528 individuals in the illustrative data set

	Modal assignment			
Classes	% correspondence	Rand (%)	Adjusted Rand (%)^a^	Drift (%)^b^
2-class^c^	90.7	83.1	65.9	116
3-class	88.6	50.6	0.3	56
4-class	68.0	77.9	54.1	−175
5-class	66.9	80.3	58.2	−326
6-class	25.1	75.1	34.6	−327
7-class	54.7	80.2	46.3	−320
8-class	45.9	79.6	42.7	−389
9-class	20.5	64.5	−0.6	−214
10-class	40.5	81.6	46.4	−335
11-class	21.3	66.5	0.4	−128

a Adjusted Rand accommodates for chance.

b Net difference in the number of individuals within the smaller classes within the AR1 model.

c For the 2-class AR1 model, the intercept variance was constrained to zero to attain convergence with non-negative variances.

### Model random structure

**Fig. 2:**
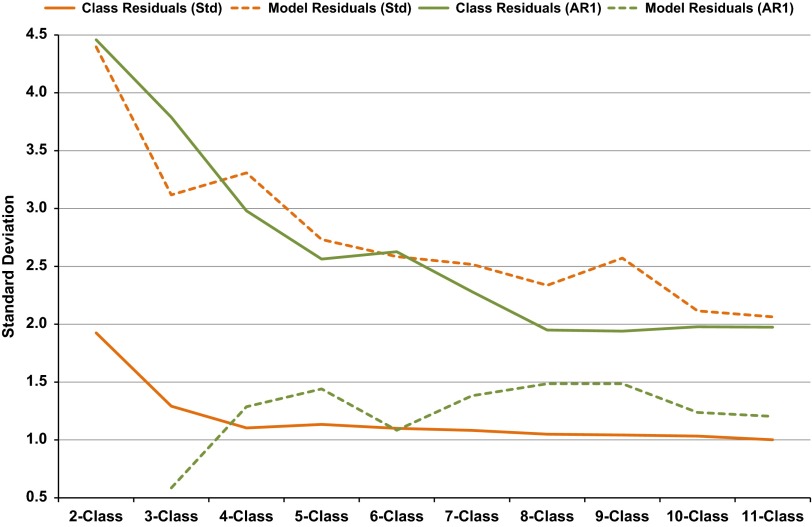
Variation in *class* trajectory intercept residual variances and *model* trajectory intercept residual variance: 10 restricted standardgrowth mixture models (GMMs; Std) and 10 restricted AR1 GMMs (AR1); for the two-class AR1 model, intercept variance was constrained to zero to attain convergence with non-negative variances.

Figure 2 summarizes the weighted mean variation of *class* trajectory intercept residual variances and the *model* trajectory intercept residual variance for the range of models considered. Class trajectory intercept residual variances were on average twice of that for models with AR1, indicating that individual trajectories were heterogeneous *within* classes when autocorrelation was accommodated explicitly. The overall model intercept residual variance was typically a third smaller for models with AR1, revealing that class trajectories were more homogeneous *between* classes in models when autocorrelation was accommodated explicitly. This illustrates the extent by which within-model/between-class and within-class trajectories are affected by the parameterization of the random structure. For these data, the AR1 parameterization elevated random intercept heterogeneity within classes, while reducing random intercept heterogeneity between classes, compared with models with a constrained variance–covariance structure and no ‘compensatory’ autocorrelation.

### Simulations

Among the simulated data sets, several models failed to converge without a non-positive definite latent variable covariance matrix, indicating either negative variances or residual variances for a latent variable or correlation greater than or equal to one between two latent variables. Modelling complex random structure is thus challenging, which is why constraining random effects to achieve convergence is so appealing. Although each of the one- to three-class restricted random effects models converged easily, this was not true for some of the four- and five-class restricted models. For the entire range of models considered, there were only nine repeated simulation data sets that were unproblematic for all models (different data sets gave rise to differing convergence problems). Table 4 summarizes the mean likelihood statistics for these data sets.

**Table 4:** Mean likelihood statistics for growth mixture models (GMMs) of nine simulated data sets

	Mean -2LL	df	Mean AIC	Mean BIC	Mean AR1(ρ)
Unrestricted					
2-class	41,877.6	21	41,919.6	42,031.6	–
Restricted standard					
1-class	42,130.9	5	42,140.9	42,167.6	–
2-class	41,980.8	11	42,002.8	42,061.5	–
3-class	41,919.5	17	41,953.5	42,044.1	–
4-class	41,892.4	23	41,938.4	42,061.0	–
5-class	41,871.5	29	41,929.5	42,084.2	–
Restricted AR1					
1-class	42,017.9	6	42,029.9	42,061.9	0.255
2-class	41,936.9	12	41,960.9	42,024.8	0.269
3-class	41,905.5	18	41,941.5	42,037.4	0.141

LL, log-likelihood; AIC, Akaike’s Information Criterion; BIC, Bayesian Information Criterion; df, degrees of freedom.

Under simulation, both the AIC and the BIC favoured the two-class unrestricted model that reflects the underlying data generation process. However, when analyses were limited to restricted models (i.e. when unrestricted models fail to converge and more parsimonious random structure is not explored), both the AIC and the BIC favoured models with more than two classes. When random effects were constrained and emergent AR structure modelled explicitly, BIC favoured the correct number of classes, but AIC did not. Likelihood statistics generally favoured models with more classes for the constrained random effects models with no AR1 structure compared with models with AR1.

When examining class composition via modal assignment there were marked differences (Table 5). Compared with the true generating model, the unrestricted GMM fared well, although it was far from perfect (adjusted Rand was just >50%). The restricted+AR1 model did well in recovering latent class membership and was very similar to the unrestricted GMM. The restricted model without AR1, on the other hand, did considerably worse (adjusted Rand of 0.2%). In contrasting the different parameterizations against each other, the unrestricted and restricted+AR1 models were very similar (class correspondence >90% and adjusted Rand of 72.2%), whereas the restricted model with no AR1 was very different from the other two parameterizations in class membership (class correspondence barely over 50% and adjusted Rand <1%).

**Table 5:** Class correspondence for two-class growth mixture models (GMMs): unrestricted random effects and restricted random effects either with or without AR1: mean (s.d.) modal assignments of 1528 individuals across the nine simulated data sets

GMM contrast made	% correspondence	Rand (%)	Adjusted Rand (%)^a^
Against true			
Unrestricted	86.1 (0.9)	76.1 (1.3)	52.2 (2.5)
Restricted AR1	86.7 (1.2)	76.9 (1.7)	53.8 (3.5)
Restricted	52.2 (1.6)	50.1 (0.1)	0.2 (0.3)
Against each other			
Unrestricted *v.* restricted AR1	92.5 (1.9)	86.1 (3.2)	72.2 (6.5)
Unrestricted *v.* restricted	52.9 (3.1)	50.3 (0.5)	0.6 (1.1)
Restricted AR1 *v*. restricted	53.3 (2.7)	50.3 (0.5)	0.6 (1.0)

a Adjusted Rand accommodates for chance.

## Discussion

In lifecourse epidemiology, identification of early-life patterns or critical periods of growth that might impact health status in later life is a rich and exciting area of research, albeit one fraught with methodological challenges.^29^ We examine the use of GMMs in the context of lifecourse evaluation of BMI and specifically reflect upon how models are parameterized in terms of their random structure. Given the increasing popularity of these methods, it is likely that many applications will adopt constraints for the random structure either as a matter of convenience to promote parsimony or out of necessity to attain model convergence. These constraints will affect the features of interest (i.e. the latent classes), creating the challenge of determining which model parameterization is ‘correct’.

Choosing the ‘correct’ model requires an understanding of the context in which data are generated to model variation correctly (i.e. to attain model parsimony without inadvertently imposing *inappropriate* constraints). The residual autocorrelation between observed values and fitted BMI trajectories is often trivial. However, within GMMs, if the variance–covariance structure is severely restricted, and as BMI exhibits greater within-person than between-person homogeneity (as with most growth measures), residual autocorrelation emerges between individual fitted trajectories and the class mean. Methods for modelling an AR structure are varied,^30^ with one being a simple autocorrelation constraint for successive measures, and another expressing the longitudinal variable as an additive function of its immediately preceding values.^31^ An example of the combined attributes of the family of LGCMs, which capture no influence of the lagged growth variable on itself, and the AR models, which do not allow for individual random effects, is the more comprehensive autoregressive latent trajectory (ALT) model.^32^ ^–^ ^34^ The simple autocorrelation constraint or the ALT model captures the emergent autocorrelation explicitly. For our growth measure, BMI, differences in model fit or overall interpretation from either approach are likely to be small, although the exact meaning of model parameters will differ slightly: the AR1 model estimates a correlation coefficient for successive growth measures, whereas the ALT model estimates a regression coefficient for each growth measure regressed on prior values. In both instances, however, the relationship (if any) among the level-1 residuals is conflated with the relationship that emerges among trajectory residuals as a consequence of the random effects constraints. Our findings suggest that for growth measures such as BMI, if the variance–covariance structure is constrained, the emergent AR structure can and should be modelled explicitly, as this leads to model improvement and more accurately captures the underlying data generation process.

In practice, selecting the ideal number of classes is informed largely by likelihood statistics but partly by model interpretation. It is clear that parsimony is desirable and the BIC is preferred as it has been shown to perform best of all such information criteria under simulation.^35^ It is also recognized that growth mixtures can be determined in the absence of genuine population heterogeneity,^36^ especially if the distribution of growth trajectories is non-normal,^37^ ^–^ ^39^ and where covariance misspecification is restrictive the estimated number of latent classes can be greater than the true number, because more are required to model the extra variability.^40^ For growth data of the kind motivating this investigation, where BMI is homogeneous over time (near linear), with considerable between-person heterogeneity, where covariance constraints are necessary, and there is no clear underlying normal distribution, one has to wonder whether there are any genuine population sub-groups or whether the GMM is merely categorizing a continuum. Given the competing factors that may lead to more classes being determined than are meaningful, it is important to pursue parsimony with GMMs while being careful to capture random structure appropriately. Striking a balance between model complexity in the random structure and parsimony, while not straightforward, is important to determine the correct number and composition of classes if the associated inferences are to be meaningful and robust.

A benefit of modelling the emergent within-class autocorrelation to compensate for the variance–covariance constraints is that a larger proportion of individuals are assigned to larger classes compared with models with no autocorrelation structure. Accounting for the random structure effectively homogenizes the larger classes and the LL statistics indicate that modelling autocorrelation in this context provides an improved model fit; though blindly adopting likelihood-based model-fit criteria may not always differentiate among plausible models.^41^

Unsurprisingly, as the number of classes increases, class correspondence decreases between models with and without the AR1 random structure. Class correspondence assumes that *relative* class sizes remain the same for all models, and hence class ranks remain the same. This is unlikely to hold. For the illustrative data set, the peculiarity of the six-class model in percentage correspondence and the 9-class and 11-class low adjusted Rand statistics were due to diagonals of some class cross-tabulations being zero, suggesting that class correspondence according to ranked class size was inappropriate; with no similar indication for the three-class model, we may only speculate that the assumption was not upheld.

There are a few limitations of this study and its findings. First, if the variance–covariance structure of a model must be constrained (e.g. to achieve convergence), the choice of alternative, more parsimonious parameterizations of the random structure is open to evaluation. For instance, the ALT model approach could be considered. We explored a serial correlation term among class trajectory residuals within each growth mixture by incorporating an ARn constraint (with *n*=1, in this instance), and the choice of ‘*n*’ is also open to evaluation. For both the illustrative and simulated data, there were only four time points and an AR1 was adequate; for more repeated measures a larger ‘*n*’ might be warranted. In general, however, we do not propose that a universal alternative approach to modelling random structure in GMMs in the presence of variance–covariance constraints is an AR1 parameterization, as it is advisable to explore a range of model options that are driven by an *a priori* understanding of the data generation processes. We note, however, that this relatively simple strategy fared well in our study.

Second, although not a problem for our illustrative data, parameterization of a simple polynomial may not always adequately capture the underlying growth trajectories. More sophisticated strategies, such as fractional polynomials splines, or freed-loading models^4^ may be needed.

Third, as often the case in longitudinal epidemiological studies (and in our illustrative data), measurement intervals may not be balanced across individuals, which may lead to inaccuracy in estimating the AR1 structure. We did not adopt a continuous time approach because this caused fewer initial starts to converge, considerably lengthened the time for each model to complete (100-fold), and required imputed ages for missing measurement occasions, without affecting our conclusions (results not shown). In general, however, one should not ignore this added complication. Depending on the data, one solution may be to fit individual curves first, and then extract a balanced set of data from those.

Fourth, parameterization of the random variation over time was constrained to be identical for every class. Relaxing this constraint may yield classes that could distinguish between more or less homogeneous individuals (a very plausible scenario), although for the illustrative data set fewer random starts attained convergence and there was no effect to our overall conclusions (results not shown).

Fifth, whether or not the random structure is fixed throughout the lifecourse is debatable. BMI generally exhibits greater individual than population homogeneity, but this might vary for different growth periods, such as the first few years of life where differences between population heterogeneity and individual heterogeneity are less. Fewer variance–covariance constraints may then be required. Consequently, each stage of the lifecourse must be examined separately for these effects, because implications of findings on growth throughout adolescence may not generalize to other periods of life. The extent of individual and population heterogeneity might vary (i.e. heteroscedasticity), and with variance–covariance constraints the emergent within-class autocorrelation might differ across the different stages of growth. Seeking to accommodate heteroscedasticity throughout different stages of the lifecourse remains an issue for future research.

Finally, it must be recognized that we have undertaken a narrow range of simulations with few parameter specifications that emulate a single observed data set. However, insights gleaned from these simulations clearly demonstrate that tampering with the random structure of GMMs for whatever motive (e.g. parsimony or to aid convergences) has substantive impact on the types of models determined. Our initial findings may inform further research so that the field can advance beyond these limitations.

## Conclusion

Where lifecourse outcomes exhibit greater within-person homogeneity (gradual changes over time) than between-person homogeneity (substantial differences between individuals), and where these outcomes are explored using GMMs with the random effects constrained (for parsimony or to attain convergence), within-class autocorrelation can emerge and should be accommodated explicitly in the model. During puberty, increased individual heterogeneity is a hallmark of adolescence and is likely to contribute to a reduced degree of within-class autocorrelation owing to greater within-person variation. Adolescence is also a time of elevated between-person heterogeneity, which is likely to contribute to an increased degree of within-class autocorrelation. The net effect of these two factors is probably population-specific; however, for our illustrative data, substantial within-class autocorrelation was induced once constraints on the random effects were introduced to achieve model convergence. Models with an autocorrelation structure were substantially different from models without yielding different class trajectories with different subjects in each class; these models more likely reflect the underlying data generation processes, according to simulations. These findings imply that failure to model random structure of growth outcomes carefully within a GMM framework can give rise to misleading models and therefore potentially erroneous inferences.

## Supplementary Material

Supplementary Material To view supplementary material for this article, please visit http://dx.doi.org/10.1017/S2040174414000130.
