# Supplementary material for: Challenges in modelling the random structure correctly in growth mixture models and the impact this has on model mixtures
Source: J Dev Orig Health Dis. 2014 Mar 3;5(3):197–205. doi: 10.1017/S2040174414000130 (PMC4098080; doi:10.1017/S2040174414000130)
Supplement: Supplementary Material — To view supplementary material for this article, please visit http://dx.doi.org/10.1017/S2040174414000130. [file S2040174414000130sup001.pdf]

http://dx.doi.org/10.1017/S2040174414000130

APPENDIX

Model specification for the observed data

The general form of the growth mixture model for the illustrative dataset was:

$$BMI_{ti} = \sum_{c=1}^{C} P(c \mid age_{ti}, sex_i, race_i)\left(\beta_{0ti}^{c} + \beta_{1i}^{c} age_{ti} + \beta_{2}^{c} age_{ti}^{2}\right)$$

where BMI_{ti} is an individual's BMI at age_{ti}, measured at time t = 1...4, for individual i = 1...1528; c is the latent class (c = 1...C), for which $P(c \mid age_{ti}, sex_i, race_i)$ is the probability that individual i is in class c given their age_{ti}, sex_i, and $race_i$; $\beta_{0ti}^{c} = \beta_{0}^{c} + e_{0ti}^{c} + \gamma_1 age_{ti} + \gamma_2 sex_i + \gamma_3 (age \cdot sex)_{ti} + \gamma_4 race_i$ is the random intercept for class c, conditioned on age, sex, age-sex interaction and race identically across all C classes; $\beta_{1i}^{c} = \beta_{1}^{c} + \gamma_5 age_{ti}$ is the velocity for class c, conditioned on age identically across all C classes; β_{0}^{c}, β_{1}^{c}, and β_{2}^{c} are the class-dependent marginal mean intercept, velocity and acceleration respectively; $e_{0ti}^{c} \sim N\left(0, \sigma_{(c)e_0}^{2}\right)$ is the class-dependent occasion-specific normal residual with zero mean and variance $\sigma_{(c)e_0}^{2}$, estimated empirically; and γ_m (m = 1...5) are class-independent conditional covariate trajectory coefficients describing the underlying population mean growth for intercept and velocity respectively. Models were expanded to include the additional autocorrelation parameter, thereby capturing more of random effects structure that could not be reflected via the constrained variance-covariance structure. With only four measurement occasions, we adopted a simple 1st order autoregressive structure (AR1). For models with AR1 the constraint $Corr\left(e_{(t)i}, e_{(t+1)i}\right) = \rho$ (t = 1 ... 3) applies identically across all C classes, else $Corr\left(e_{pi}, e_{qi}\right) \equiv 0$ ($\forall\ p \neq q$). Example M-Plus code and associated model path diagram are given in Figures A1 & A2.

http://dx.doi.org/10.1017/S2040174414000130

Figure A1: Example M-Plus code for 2-class AR1 GMM with a bootstrapped likelihood ratio test, for 2000 random starts and 200 fully evaluated starts.

```
TITLE:   Analysis of BMI
DATA:
    FILE IS bmi.csv; LISTWISE = OFF;
VARIABLE:
    NAMES ARE  sch id sex race age1 age2 age3 age4 bmi1 bmi2 bmi3 bmi4
               cdc1 cdc2 cdc3 cdc4 wgt1 wgt2 wgt3 wgt4 hgt1 hgt2 hgt3 hgt4
               post1 post2 post3 post4 srcesd1 srcesd2 srcesd3 srcesd4
               sranx2 sranx3 sranx4 ses1 ses2 ses3 ses4 edu;
    MISSING ARE ALL (-99);
    USEVARIABLES = sex bmi1-bmi4 race agec agesex sexrace sexm racem agesexm;
    AUXILIARY = sex (e) race (e) age1 (e) agesex (e) sexrace (e) cesd1-cesd4 (e)
               anx2-anx4 (e) srcesd1-srcesd4 (e) sranx2-sranx4 (e) post1 (e)
               ses1-ses4 (e) edu (e);
    CLASSES = c(2);
DEFINE:
    agec = age1-1.440695; sexm = sex; racem = race; agesexm = agec*sex;
    agesex = agec*sex; sexrace = sex*race;   ! generated to test only
ANALYSIS:
    TYPE = MIXTURE; LRTBOOTSTRAP;
    MITERATIONS = 2000; STITERATIONS = 50; PROCESSORS = 8;
    MODEL = NOCOVARIANCES;
    LRTSTARTS = 2000 200 2000 200;
    STARTS = 2000 200;
MODEL:
    %OVERALL%
        Int Vel Acc | bmi1@-1.5 bmi2@-0.5 bmi3@0.5 bmi4@1.5 ;
        Vel-Acc@0;
        Int ON agec sexm agesexm racem;
        Vel ON agec;
    %c#1%
        bmi1-bmi4 (resvar1);
        bmi1-bmi3 PWITH bmi2-bmi4 (p11);
        bmi1-bmi2 PWITH bmi3-bmi4 (p21);
        bmi1 WITH bmi4 (p31);
    %c#2%
        bmi1-bmi4 (resvar2);
        bmi1-bmi3 PWITH bmi2-bmi4 (p12);
        bmi1-bmi2 PWITH bmi3-bmi4 (p22);
        bmi1 WITH bmi4 (p32);
    MODEL CONSTRAINT:
        NEW (corr);
        p11 = resvar1*corr ; p21 = resvar1*corr**2 ; p31 = resvar1*corr**3;
        p12 = resvar2*corr ; p22 = resvar2*corr**2 ; p32 = resvar2*corr**3;
OUTPUT:
    STAND; TECH14;
SAVEDATA:
    FILE = bmi2.dat; SAVE = CPROBABILITIES;
PLOT:
    TYPE IS PLOT3;
    SERIES IS bmi1-bmi4(Vel);
```

http://dx.doi.org/10.1017/S2040174414000130

Figure A2: Model Path Diagram

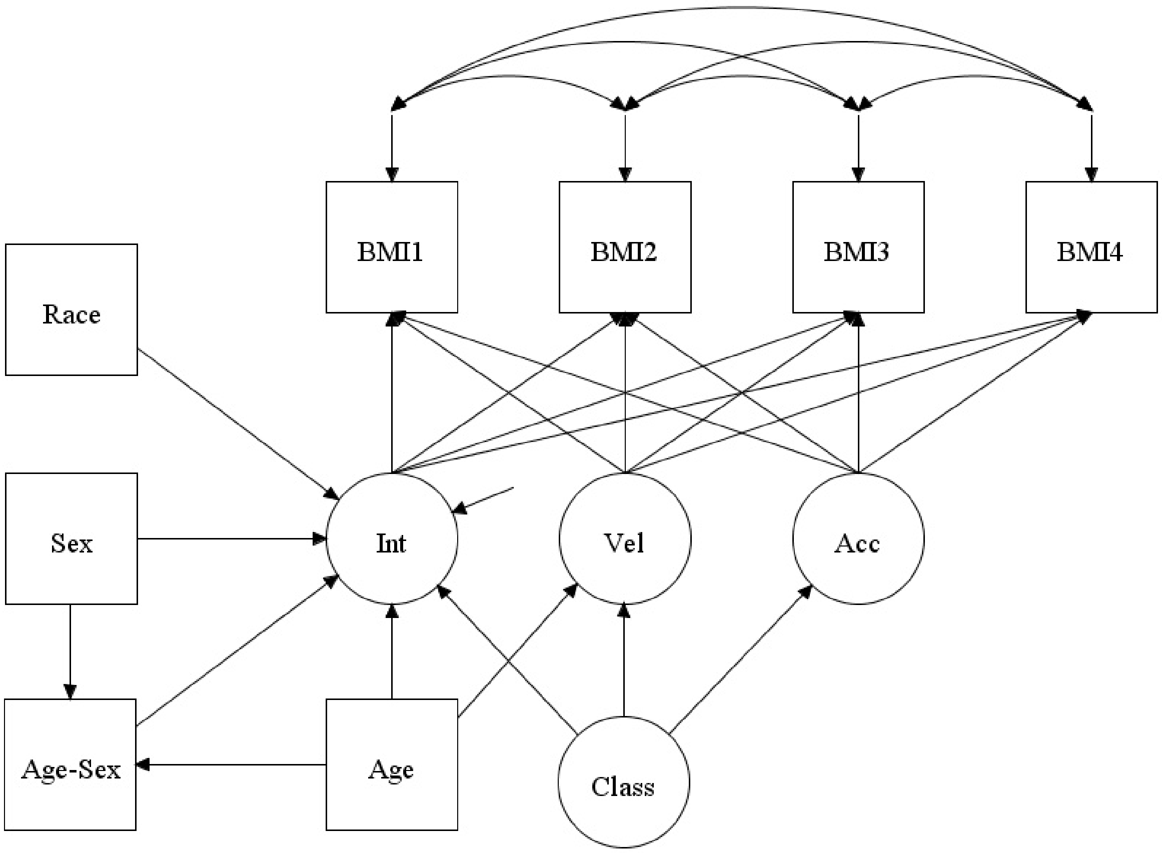

Model specification for the simulated data

We simulated two BMI latent growth curves following quadratic variation in time. Fixed-parameter differences were in the BMI mean intercept only and random effects were different across classes. One hundred replicate datasets were generated, each comprising 1528 individuals at four time points (-1.5, -0.5, 0.5, and 1.5), with BMI mean intercepts 25.0 kg.m^{-2} in class 1 and 15.0 kg.m^{-2} in class 2; both classes had identical mean velocity (5.0 kg.m^{-2}.yr^{-1}), mean acceleration (1.0 kg.m^{-2}.yr^{-2}) and BMI intercept (10.0 kg^{2}.m^{-4}.yr^{-2}), velocity (5.0 kg^{2}.m^{-4}.yr^{-2}) and acceleration (1,0 kg^{2}.m^{-4}.yr^{-2}) variances. In class 1, the BMI intercept-velocity covariance was 0.2 (correlation = 0.028) and intercept-acceleration covariance was 0.1 (correlation = 0.032), whereas in class 2 these were -0.2 (correlation = -0.028) and -0.1 (correlation = -0.032), respectively, and for both classes the velocity-acceleration covariance was 0.05 (correlation = 0.022). The intercept, velocity and acceleration were not dependent on any other factors. These hypothetical datasets do not therefore emulate the illustrative

http://dx.doi.org/10.1017/S2040174414000130

study data exactly, as no attempt was made to simulate emulate ages at each time point or age-related effects (such as individual growth spurts). This should not affect any inferences made regarding differences in model parameterization. The simulations broadly capture the concept of a near linear change in BMI over time, with large heterogeneity between individuals and small heterogeneity within individuals between measurement occasions, as per the study data.
